# Long-term effects of bifrontal transcranial alternating current stimulation on verbal working memory and episodic memory in young healthy adults

**DOI:** 10.1038/s41598-025-29148-2

**Published:** 2025-12-29

**Authors:** Haim Raviv, Nira Mashal, Oded Meiron

**Affiliations:** Faculty of Education, Bar-Ilan University, Ramat Gan, 5290002 Israel

**Keywords:** Prefrontal cortex, Frontal theta power, Verbal working memory, Transcranial alternating current stimulation (tACS), Neuroscience, Psychology, Psychology

## Abstract

The current randomized controlled trial (RCT) examined both immediate and long-term effects of five consecutive bifrontal tACS sessions on verbal WM functioning in 30 healthy adult participants. WM performance and event-related quantitative electroencephalography (qEEG) parameters (mean power of frontal theta oscillations during three different WM events) were noted at baseline, immediately after tACS intervention, and at four-week post intervention. In comparison to the sham-tACS group, WM performance in active tACS group was improved following a four-week post-intervention period across all WM events, but not immediately after tACS intervention. Left prefrontal theta power in the active group decreased immediately after the intervention (in comparison to baseline) and remained low at four weeks, while the sham group returned to high theta-power baseline levels. Additionally, at four weeks post-intervention, participants in the active group demonstrated higher episodic memory accuracy compared to the sham group, which was associated with lower frontal theta power during WM encoding events. These findings are consistent with the hypothesized long-term plasticity effects. The current results highlight the role of event-related prefrontal theta power modulation in enhancing verbal WM and episodic memory retrieval in humans acutely, and over time.

## Introduction

Working memory (WM) is an essential cognitive function that supports many everyday tasks that involve attentional control of information maintenance in WM which significantly impact decision-making to problem-solving behaviors in humans^1^. Critically, WM retention mechanisms are highly active during temporary storage and manipulation of information, which is supported by a distributed neural-network spanning across frontal and parietal cortical regions^2,3^. Importantly, oscillatory activity in the theta frequency band (4–8 Hz) has been consistently implicated as a key neural signature of WM processes^4,5^. Source localization of WM-related frontal midline theta activity and fMRI findings indicated the anterior cingulate (ACC), medial prefrontal cortex (mPFC), dorsolateral prefrontal cortex (DLPFC), middle frontal gyrus, and the hippocampus are involved in the generation of theta oscillations across a distributed cortical-limbic WM networks^6^. Research using EEG and MEG techniques has demonstrated that higher theta power was associated with higher cognitive demands during WM tasks^7,8^.

Given the role of theta oscillations in WM, transcranial alternating current stimulation (tACS) has been explored as a method for modulating oscillatory neural activity to improve WM performance. tACS is a noninvasive technique that applies weak sinusoidal electrical currents to synchronize with endogenous brain rhythms, potentially enhancing functional connectivity and executive WM performance^5,9,10^. Studies show that tACS targeting specific frequency bands, such as theta, can influence cognitive functions associated with frontal theta oscillations, improving WM performance and altering theta power dynamics^10^.

A widely used paradigm for assessing WM performance in humans is the *n*-back task, as it systematically engages core WM processes, including encoding, maintaining, and retrieving information over short time intervals^11–14^. Encoding involves sensory processing and temporarily storing target information from recently presented stimuli. WM retention intervals reflect *active maintenance* of information over time versus disregarding non-target intervening trials (representing an ongoing source of interference requiring active inhibition of irrelevant stimuli). Successful encoding and maintenance are likely to result in better WM performance and improved executive attentional control of information accessible in WM storage^13,15,16^.

Studies examining the effects of single-session theta-rhythm tACS protocols or combined theta/alpha theta/gamma tACS protocols on WM performance in healthy young adults have yielded mixed results. Some research has reported significant improvements, particularly with frontoparietal cortex (e.g^5,17–20^. but see Paßmann et al.^21^ and Vosskuhl et al.^22^, who found effects limited to post-encoding or short-term memory, with no improvements during online performance or in working memory). Studies targeting the parietal cortex have likewise reported a beneficial effects on WM^23,24^. Conversely, other studies, especially those applying tACS exclusively over the frontal cortex, tend to report partial improvements in WM performance^10,25–27^ or no significant effects^23,24,28^.

Research exploring multiple-session tACS effects on WM over short periods (2 to 5 days) is relatively rare. Among the few, Möller et al.^29^ found that five daily sessions of parietal tACS impaired visuospatial WM, while Nomura et al.^30^ demonstrated that two sessions of gamma-tACS over the left PFC improved long-term memory retention up to seven days post-intervention. Other studies, like Murray et al.^31^, observed impaired verbal recall performance during theta-tACS targeting the left temporal lobe. In contrast, Diedrich et al.^32^ reported significant improvements in visuospatial WM after 16 sessions of theta-gamma tACS targeting the DLPFC over six weeks.

The second study, by Nomura et al.^30^, aimed to explore the lasting effects of gamma-frequency tACS (60 Hz) on memory retention by applying stimulation over the left prefrontal cortex (PFC). The study involved 2 sessions of stimulation over F3 (with reference on left wrist) during a learning task of words on day 1 and a recognition task on day 2, with memory retention assessed through additional recognition tasks on days 1, 2, and 7. The findings revealed significant improvements in long-term memory retention in the tACS group compared to the sham group, indicating that gamma-frequency tACS over the left PFC can induce enduring neuroplastic changes that enhance memory retention, as evidenced by enhanced retention on day 7.

The acute effects of multi-session tACS on recall performance were also investigated by Murray et al.^31^, who explored the effects of theta-frequency tACS (6 Hz) on verbal recall, targeting the left temporal lobe. Theta tACS was delivered during a list-learning task in three separate sessions as part of a randomized sham-controlled design investigating healthy adults. Results revealed that tACS impaired recall performance, with participants recalling significantly fewer words during tACS sessions compared to sham. Additionally, tACS led to more intrusion and repetition errors than sham stimulation. These findings suggest that theta tACS, when applied to the left temporal lobe, may negatively impact recall processes.

In contrast, focusing on the dorsolateral prefrontal cortex (DLPFC), Diedrich et al.^32^ investigated the effects of repeated theta–gamma (6 Hz, 80 Hz) tACS in older adults. In this randomized, sham-controlled trial, 77 participants received sixteen sessions of stimulation over six weeks, with electrodes placed at F3 (anode) and F4 (cathode). During each session, participants performed online 1- and 2-back tasks. Both the active and sham groups improved across sessions on the 2-back task, but the improvement was greater in the active group. Importantly, the study examined only online performance effects; no post-intervention or long-term follow-up WM assessments were conducted.

In response to the mixed findings from single and multi-session tACS studies and the positive results from long-term multi-session tACS stimulation over the DLPFC, the current study aims to systematically investigate the behavioral and neural signatures of multi-session tACS intervention effects targeting the DLPFC in healthy humans. The previously reported studies have primarily focused on visuo-spatial WM functioning, leaving verbal WM largely unexplored. While prior research on verbal WM processes has highlighted the potential of single-session theta-tACS to modulate theta activity^10^, the effects of consecutive, multi-session tACS on both immediate and long-term verbal WM performance remain underexplored.

Therefore, primary objectives of the current study are: (1) to investigate the acute (immediate) and long-term effects (4 weeks post intervention) of multi-session bifrontal (targeting the DLPFC) theta (6 Hz) tACS over a period of five consecutive days on verbal WM performance and frontal theta oscillations; (2) to evaluate episodic memory performance and long-term neuroplasticity effects (changes in mean theta power) at 4 weeks post intervention, comparing tACS to sham; and (3) to examine the associations between changes in frontal theta power at each time point (baseline, immediate after intervention at day 5, and at 4 weeks post intervention) and changes in WM performance (accuracy and reaction times). Based on Meiron and Lavidor^10^, who found an effect of single-session tACS over the DLPFC on verbal WM performance, and Diedrich et al.^32^, who demonstrated both acute and long-term effects of multi-session tACS on other WM performances, we hypothesized that immediately after a five-session tACS intervention, as well as four weeks post-intervention, effects would be observed on verbal WM performance, theta activity, and the associations between theta activity and performance.

## Methods

### Participants

A total of 30 participants, all healthy native Hebrew speakers with normal or corrected-to-normal vision, were included in the study. This sample size is consistent with prior research in the field. For example, Meiron & Lavidor^10^ conducted a tACS study with 24 participants, Mashal & Metzuyanim‑Gorelick^33^ conducted a tDCS study with 25 participants while Diedrich et al.^32^ included 35 participants. Given that our sample size falls between these studies, it is well within the range commonly used in similar experimental designs. Participants were randomly assigned to one of two groups in a between-subject design: the active stimulation group (*n* = 15) and the sham group (*n* = 15). Both groups were balanced in terms of gender distribution, with each consisting of 9 women and 6 men, in terms of age (t(23.35) = 1.06, *p* =.30, *d* = 0.39), Digit Span Test forward scores (t(27.86) = 0.85, *p* =.39, *d* = 0.31), Digit Span Test backward scores (t(27.92) = 0.57, *p* =.57, *d* = 0.21) and semantic fluency scores (t(27.31) = 1.16, *p* =.25, *d* = 0.43) at baseline (see Table 1). Thus, both groups were balanced in terms of age, gender distribution, cognitive performance, and verbal ability at baseline, prior to tACS intervention. All participants were right-handed.

**Table 1:** Descriptive statistics for demographic variables (Means and standard Deviations).

	Active		Sham	
	Mean	SD	Mean	SD
Age	22.9	2.0	24.0	3.3
Digit Span Forward	9.40	2.19	10.07	2.05
Digit Span Backward	6.47	2.16	6.93	2.28
Fluency Test	62.27	14.52	68.0	12.37

Participants self-reported that they were healthy and did not have any medical conditions (neurological or psychiatric history) or other factors (unstable medical condition) that might interfere with the study. Specifically, they reported no history of epilepsy, no metal objects in their head, no pacemakers, no drug or alcohol addiction, and no use of psychiatric medications. The study was approved by the Ethics Committee of the Faculty of Education, Bar-Ilan University, Ramat Gan, Israel. License number: 17. All methods were performed in accordance with the relevant guidelines and regulations.

### Materials

#### Digit span task

The digit span (DS) task, consisting of both forward and backward recall components, is a standardized measure included in the Wechsler Adult Intelligence Scale (WAIS)^34^. In this study, the task was administered orally by the experimenter, with digit sequences read aloud in gradual WM-load increments at each new trial. Each sequence ranged in length from three to nine digits, with digits spoken at a pace of one digit per second. For forward recall, participants were instructed to repeat the digits in the exact order in which they were presented. For backward recall, participants were instructed to recall the reverse sequence of digits. Task progression was monitored by the experimenter, increasing sequence length after every correct response. This task is considered a reliable neuropsychological assessment of basic working memory functioning in adults^34^.

#### Semantic fluency test

The fluency test is designed to evaluate oral verbal fluency and executive functioning, particularly sensitive to neural activity in language production areas, and directly related to semantic memory retrieval and cognitive flexibility^35^. It consists of a semantic fluency task, where participants vocally generate words belonging to a specific category (e.g., animals, fruits) within a 60-second time frame. The test measures the speed and accuracy of semantic retrieval and is particularly useful for assessing language-based executive functions in healthy individuals. It provides a reliable baseline measure of abilities such as verbal memory fluency, semantic memory, and cognitive flexibility^36^.

### Verbal n-back task

**Fig. 1:**
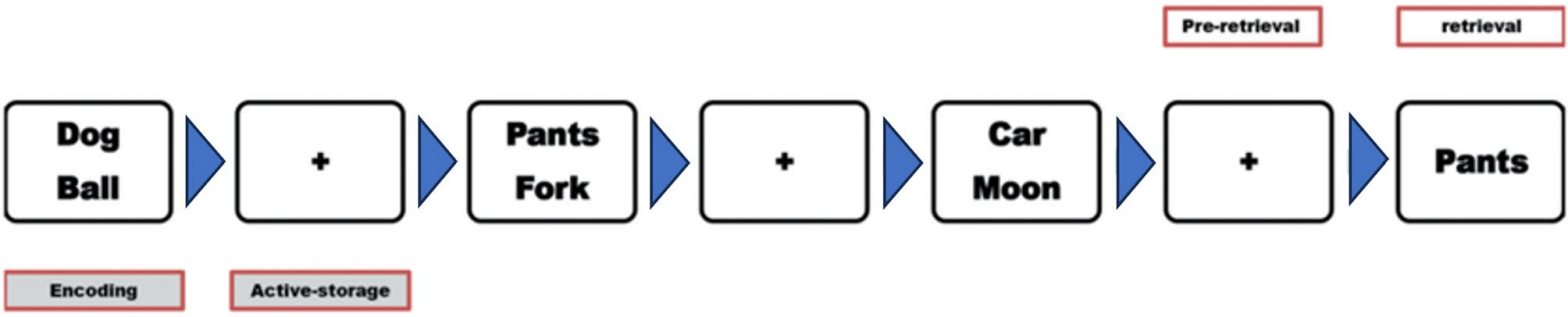
Verbal working-memory n-Back task. Illustration displays a 2-back task trial including three encoding events (word-pair squares, 2000 ms intervals), two active-storage events (squares with “+”, 2000 ms intervals), and one retrieval event (response-display, 2000 ms interval). Participants were instructed to respond as fast as possible by key-press if the single word in the retrieval event was part of a word-pair from an encoding event two displays earlier (Pants-Fork). In the current illustration (3 word-pair display), the participant was expected to correctly respond to the retrieval word “Pants”^14^.

In this task, participants were presented with a series of word-pair displays on a PC-screen and were instructed to determine whether a single word, presented later, had appeared in the display shown two steps earlier (2-Back task). Each trial began with a word-pair display, where participants studied pairs of words on the screen for 2000 ms during an encoding interval (see Fig. 1). These encoding intervals were followed by 2000 ms interstimulus intervals (ISIs), during which participants focused on fixation cues at the center of the screen while *actively maintaining* the recently encoded word-pairs in working memory (active storage interval). After the first word-pair display, each trial could continue with quasi-randomly determined 1 to 4 additional word-pair displays (resulting in a total of 2 to 5 consecutive word-pair displays), each followed by a 2000 ms ISI for active storage. An individual trial is concluded with a retrieval interval, during which a single word (the potential target) appears at the center of the screen. Participants were required to respond by pressing a key if the presented word matched one from a pair shown during the encoding interval two displays earlier (2-Back). If the word did not match, no response was required. 50% of the response displays were target-words taken from a two-back display (expected key-press response) and the other 50% were taken from non-target positions within a trial (e.g., one-Back or three-Back positions, no response expected). The task consisted of a total of 60 trials, divided as follows: 20 trials with a sequence of 2 word-pair displays, 20 trials with 3 word-pair displays, 10 trials with 4 word-pair displays, and 10 trials with 5 word-pair displays that precede the single word (retrieval interval). These trials were further organized into 5 balanced blocks. The variables measured were the reaction times (RTs) for target words and the accuracy of target recognition (which included both correct responses and correct avoidances). The 2-Back task lasted approximately 25 min, during which EEG was continuously recorded. The setup and baseline calibration prior to the task added ~ 5 min, making the total recording time ~ 30 min per participant. Each trial block lasted about 5 min, with breaks of 30 s. between. Electrode sponges were initially soaked in saline solution, and due to the relatively short recording period, no re-soaking was required during the sessions to maintain good contact and signal quality.

Adapted from Meiron & Lavidor^10,12^, modified n-Back task, the stimulus-displays presented 60 different pairs of Hebrew word-*nouns*. Each word within a pair was selected from distinct semantic categories (e.g., plants and animals) and consisted of one to two syllables. The words represent everyday objects that are easily activated and retrieved from long-term memory. Meiron & Lavidor’s free recall test confirmed that all target word-pairs were equally accessible, demonstrating inter-item associative strength homogeneity^37^.

#### Explicit recall task

The explicit recall task^38^ was administered at the 3rd time point (4 weeks post intervention), five minutes after the verbal *n*-back task, to evaluate participants’ ability to explicitly retrieve specific target stimuli from a previous event. Participants were instructed to orally recall, without prompts, all the single-words they recognized as targets during the *n*-back task. Notably, participants were not informed in advance about the recall task, ensuring that their responses during the *n*-back task reflected natural working memory engagement rather than intentional encoding for later recall. This design aimed to measure the extent to which target words were successfully encoded during the *n*-back task and subsequently explicitly retrieved from episodic memory after a delay. Administering this task at the 3rd time point provides a unique opportunity to assess long-term memory consolidation processes in episodic memory, particularly in relation to the period of four weeks after tACS intervention.

### tACS

Participants were randomly assigned to two different tACS conditions, (1) Active bilateral DLPFC tACS, and (2) bilateral DLPFC sham tACS. Following scalp measurements, tACS was delivered by a battery-driven Soterix Medical DC-stimulator (Mini-CT, Soterix Medical Inc., USA) using a pair of conductive-rubber electrodes placed in saline-soaked synthetic sponge. Electrode sizes were 5 × 5 cm. All electrode positions were in accordance with the international 10–20 EEG system. The tACS bifrontal montage for the active bilateral prefrontal condition was placed over the left and right DLPFC (corresponding to F3 and F4 EEG electrode locations, respectively). The sham stimulation montage was the same as the active bilateral DLPFC montage with only 60 s of active tACS spanning over the first and last 30 s of the sham-tACS session (360 cycles). Alternating current stimulation was sinusoidal with 2 mA peak-to-peak current amplitude. Bipolar alternating current frequency was set at 6 Hz. In the active tACS group, the delivery of 7200 AC cycles was divided to two consecutive stimulation-sessions of 3600 cycles, with 120 s inter-session interval without stimulation between them to allow manual replacement of batteries (20 min of active tACS over a period of 22 min). Notably, longer daily intersession intervals have been used in non-invasive brain stimulation studies reporting benefits for WM in schizophrenia^39^ and cognitive status in dementia^40^. The present study in healthy participants applied a short intersession interval not previously used in WM protocols, although 60-second intervals have been tested for multitasking performance^41^. Current was ramped up and down for 30 s at onset and termination of tACS sessions and impedance was kept below or equal to 5 kΩ.

### EEG recording and preprocessing

The study utilized the EMOTIV wireless EEG system (EPOC+) with 14 saline-soaked felt electrodes placed according to the 10–20 international system and reference electrodes at P3 (global reference) and P4 (noise cancellation). The CMS P3 electrode served as the global reference, and the DRL P4 electrode was used for noise cancellation, ensuring reliable recordings comparable to medical-grade systems. Signals were sampled at 2048 Hz, down sampled to 128 Hz, and filtered with a 50 Hz notch filter and a 64 Hz low-pass filter. Data epochs for specific tasks ranged from 1 to 2 s, and pre-processing included mean removal, application of a Hanning window, and calculation of Power Spectral Densities (PSDs) using Welch’s algorithm in 1 Hz bins. To minimize contamination, artifact rejection combined Emotiv’s automatic preprocessing algorithms with additional quality checks. Epochs containing eye blinks or horizontal eye movements, sudden voltage steps, or excessive noise (defined as spectral power > 20 µV²/Hz in the 0.5–40 Hz range) were automatically discarded. Prefrontal channels AF3 and AF4, which consistently captured eye-movement artifacts, were excluded across participants. Channels with persistently poor electrode contact, as indicated by Emotiv’s Contact Quality index, were also removed. Finally, clean data segments were transformed via Fast Fourier Transform (FFT) to obtain mean squared absolute power values.

### Procedure

**Fig. 2:**
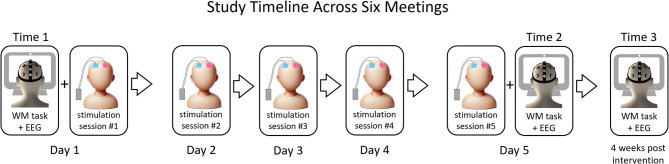
The flowchart outlines the six-meeting study timeline. On day 1 (Baseline), participants completed a baseline working memory task with EEG (Time 1), and then received the first session of tACS or sham stimulation. Over days 2–4 post-baseline, participants received sessions 2–4 of tACS or sham, depending on their group assignment. On day 5 post-baseline, participants completed their fifth and final stimulation session, followed by the WM task with EEG (Time 2) to assess short-term effects. All five meetings were held across five consecutive weekdays. Finally, in the sixth meeting (4 weeks post-intervention), participants completed the WM task with EEG again (Time 3) to evaluate long-term effects and performed an explicit recall task to assess memory retention.

Each participant attended six meetings over the course of the study (see Fig. 2). The initial meeting included obtaining written informed consent followed by gathering demographic and personal information, as approved by the Ethics Committee of the Faculty of Education at Bar-Ilan University. Subsequently, participants completed the pre-tACS verbal WM task (Time 1) during online EEG acquisition, and immediately were assigned to either active or sham tACS condition to receive the first out of five consecutive daily tACS sessions (session 1). In sessions 1 through 5 (across five consecutive weekdays), participants underwent tACS or sham stimulation, depending on their assigned condition. On the fifth day of tACS intervention, participants received their final (fifth) stimulation session. Immediately after the final tACS session they completed again the verbal WM task while their EEG was being recorded, to assess the immediate effects of tACS intervention on WM performance and WM-related brain activity versus baseline (Time 2). On the final meeting, 4 weeks post intervention (Time 3), participants completed the WM task again with online EEG acquisition to assess tACS after-effects on WM functioning. Additionally, at Time 3, after the WM task, they performed an explicit recall task designed to evaluate semantic memory retention and episodic memory retrieval of targets information from long-term memory. The between-group comparison utilized a randomized, double-blind sham-controlled design, ensuring that neither the participants nor the researchers administering the sessions were aware of the assigned condition (active or sham).

### Statistical analysis

A multi-level analysis method was employed, utilizing linear mixed models (LMM) based on single-trial, longitudinal data, to evaluate the combined effect of group (active vs. sham) and time (baseline, 5 days post-baseline, and 4 weeks post intervention) on reaction times, accuracy, and qEEG features (e.g., mean frontal theta power). The use of single-trial data allowed for a more detailed analysis of the time-based changes of functional qEEG variables and behavioral responses, reducing variability introduced by trial averaging and enabling the detection of subtle within-subject effects. qEEG analyses focused on noting changes in mean power of theta oscillations (4–7 Hz), with F3 as the main electrode of interest (superior to the left dorsolateral prefrontal cortex). The models were fitted using Restricted Maximum Likelihood (REML) estimation. To obtain accurate estimates of fixed effects and their standard errors, the Kenward-Roger approximation was applied. This method provides adjusted degrees of freedom, resulting in more reliable p-values for the fixed effects, especially in the presence of small sample sizes^42^. The data were analyzed using R and the package *lme4*^43^. The models included group (between-subjects), time (within-subjects), and their interaction as fixed factors. Notably, random intercepts were incorporated in the models for both participants and task trials to account for individual differences in baseline performance and variability across items, ensuring that the hierarchical structure of the data was appropriately modeled.

The LMM method was used to analyze the reaction times (RTs) and mean frequency spectral power values (EEG). These variables showed acceptable distributional properties, with skewness values below 1.9. Trials with incorrect responses were excluded from the analysis (14.4%) because accuracy was essential for interpreting the cognitive processes of the task. Trials with RTs exceeding ± 2 SDs (7.6%) were also excluded, following a standard procedure to remove extreme outliers that may reflect attentional lapses rather than genuine task performance^44^. The basic structure of the hierarchical model equation was specified as: (DV ~ Group * Time + (1 | Participant) + (1 | TrialType)). Accuracy, being binomial, was analyzed using a generalized linear mixed-effects model (GLMM) with a binomial distribution and a logit link function, with significance determined by z-tests for the fixed effects. Although the data were negatively skewed (−2.3), as is typical for binomial outcomes, the GLMM appropriately accounts for the bounded and non-normal distribution of such data, ensuring valid statistical inference^43,45^. Sessions with less than 50% accuracy were excluded from the analysis (2.4% of all trials), following common practice in N-back and related paradigms to ensure that only data from participants who engaged reliably with the task were analyzed^46,47^. The basic structure of the hierarchical model equation was: (DV ~ Group * Time + (1 | Participant) + (1 | TrialType),* binomial)*. Bonferroni corrections were applied for post hoc comparisons, and statistical significance was set at a p-value threshold of 0.05.

To investigate the relationship between behavioral results and EEG findings, we analyzed correlations between changes in theta power and task performance across the three time points of the WM task: time 1 (baseline), time 2 (5 days post-baseline), and time 3 (4 weeks post intervention). Differences between these time points were calculated to capture changes in theta power, and the correlations between these deltas (Δs) in theta and corresponding Δs in task performance were examined to assess whether tACS-induced modulations in theta power are functionally associated with behavioral outcomes. For RTs, Δs were calculated based on single-trial data to capture fine-grained temporal variations, whereas for accuracy, Δs were computed after aggregating the data per participant, as the raw accuracy data is binomial. EEG Δs were calculated using the same respective methods as those used for RTs and accuracy.

During the EEG data cleaning and correction, a few EEG files were too noisy, resulting in missing data in specific participants. However, the analysis was conducted using REML estimation, which is well-suited for handling incomplete and unbalanced data^48,49^. In the tACS group, data was missing for 2 participants at Time 1, 1 participant at Time 2, and 1 participant at Time 3. In the sham group, data were unavailable for 1 participant at Time 1, and 2 participants at Time 3.

## Results

The estimated means, standard errors (SEs), and confidence intervals (CIs) for reaction times and qEEG data were calculated using LMM, while accuracy was analyzed using GLMM. These approaches allowed for more accurate estimation of effects by accounting for random effects.

### Behavioral results

The linear mixed-effects models were dummy-coded, with the sham group at Time 1 specified as the reference condition. For RTs, the main effect of group was not significant (*β* = 32.28 ms, *SE* = 53.63, *t*(34.14) = 0.60, *p* =.55) and indicating that at Time 1 (baseline), the active group did not differ from the sham group. The main effects of Time 2 (*β* = 11.50 ms, *SE* = 18.87, *t*(1961.43) = 0.61, *p* =.54) and Time 3 (*β* = 25.67 ms, *SE* = 19.33, *t*(1968.17) = 1.33, *p* =.18) were also not significant, suggesting no reliable change in RT across time.

**Fig. 3:**
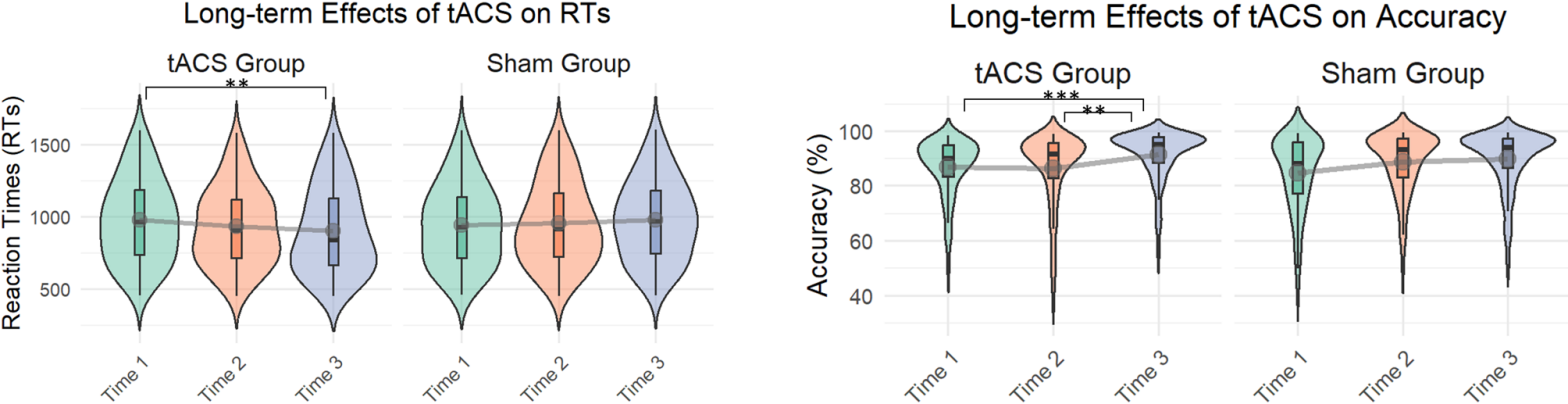
Estimated means of Reaction Times (RTs) and Accuracy (ACC) across groups at three time points: Baseline (Time 1), 5 days post-baseline (Time 2), and 4 weeks post intervention (Time 3).

The critical findings emerge in the group × time interactions. The group × Time 2 interaction showed a relative improvement in the active group compared to sham (*β* = − 52.69, *SE* = 27.08, *t*(1964.65) = − 1.95, *p* =.052). By Time 3, this effect strengthened, with the active group showing a significantly greater improvement compared to sham (*β* = − 93.25, *SE* = 27.23, *t*(1967.78) = − 3.43, *p* <.001). Pairwise comparisons within the active group, with Bonferroni correction across time, revealed that RTs at Time 3 were significantly faster than at Time 1 (β = 67.6 ms, SE = 19.2, t(1967) = 3.52, *p* =.0013). The comparison between Time 1 and Time 2 did not reach significance (β = 41.2 ms, SE = 19.4, t(1968) = 2.12, *p* =.10), nor did the difference between Time 2 and Time 3 (β = 26.4 ms, SE = 19.0, t(1969) = 1.39, *p* =.50) (Tables 2 and 3; Fig. 3).

For accuracy, the main effect of group was not significant (*β* = − 0.31, *SE* = 0.45, *z* = − 0.68, *p* =.50) and showed that at Time 1 (baseline), the active group did not differ from the sham group. Within the sham group, the main effect of Time 2 (*β* = 0.33, *SE* = 0.16, *z* = 2.09, *p* =.037) and Time 3 (*β* = 0.35, *SE* = 0.17, *z* = 2.09, *p* =.037) demonstrated higher accuracy compared to Time 1.

Turning to the group × time interactions, no significant difference emerged between active and sham at Time 2 (*β* = − 0.07, *SE* = 0.23, *z* = − 0.31, *p* =.75). However, at Time 3 (group × Time 3) the active group demonstrated a significant relative improvement in accuracy compared with sham (*β* = 0.61, *SE* = 0.25, *z* = 2.48, *p* =.013). Pairwise comparisons within the active group with Bonferroni correction across time revealed no significant difference in accuracy between Time 1 and Time 2 (odds ratio (OR) = 0.77, SE = 0.13, z = − 1.55, *p* =.36). Accuracy was, however, significantly higher at Time 3 compared with Time 1 (OR = 0.38, SE = 0.07, z = − 5.27, *p* <.001) and also higher at Time 3 compared with Time 2 (OR = 0.50, SE = 0.09, z = − 4.06, *p* <.001) (Tables 2 and 3; Fig. 3).

**Table 2:** Estimated Means, standard Errors, and confidence intervals for reaction times (RTs) and accuracy (ACC) at 3 time points (Baseline, 5 days Post-Baseline, and 4 weeks post intervention) across Groups.

	Group	Time	E.mean	SE	df	Lower.CL	Upper.CL
RT	Active	Time 1	983	38.8	37.8	904	1061
		Time 2	941	38.6	37.1	863	1020
		Time 3	915	38.5	36.7	837	993
	Sham	Time 1	951	38.7	37.6	872	1029
		Time 2	965	38.4	36.4	887	1043
		Time 3	981	38.7	37.6	902	1059
ACC	Active	Time 1	87.8	3.29	-	79.7	92.9
		Time 2	90.3	2.7	-	83.5	94.4
		Time 3	94.9	1.53	-	90.9	97.2
	Sham	Time 1	89.1	2.97	-	81.8	93.7
		Time 2	92.1	2.26	-	86.3	95.5
		Time 3	92.3	2.23	-	86.6	95.6

**Table 3:** Fixed effects for RT and ACC, with estimates, odds ratio (OR), marginal R² (m. R²) and conditional R² (c. R²).

	Fixed effects	Estimate	Std. Error	df	t value	Pr(>|t|)	m. *R*²	c. *R*²
	(Intercept)	950.35	38.97	37.63	24.388	< 0.001 ***		
RT	Active	32.28	53.63	34.14	0.602	0.551127	0.261	0.006
	Time2	11.5	18.87	1961.43	0.61	0.542171		
	Time3	25.67	19.33	1968.17	1.328	0.184219		
	Active X Time2	−52.69	27.08	1964.65	−1.946	0.051822		
	Active X Time3	−93.25	27.23	1967.78	−3.425	< 0.001 ***		
	Fixed effects	OR	Std. Error	-	z val.	Pr(>|t|)	0.322	0.020
	(Intercept)	2.28082	0.32905	-	6.931	< 0.001 ***		
ACC	Active	−0.30828	0.45346	-	−0.68	0.4966		
	Time2	0.33019	0.15791	-	2.091	0.0365 *		
	Time3	0.34718	0.16608	-	2.09	0.0366 *		
	Active X Time2	−0.07207	0.22926	-	−0.314	0.7533		
	Active X Time3	0.61153	0.24631	-	2.483	0.0130 *		

For the Explicit recall task, conducted at Time 3 (4 weeks post intervention), the percentage of correctly recalled words was calculated for each participant. A t-test revealed a significant effect favoring the active group (t(26) = 1.96, *p* =.03, *d* = 0.74), indicating higher retrieval rates compared to the sham group. Participants in the active group demonstrated a mean recall rate of 39.28% (SD = 22.27), which was higher than the sham group’s mean recall rate of 23.80% (SD = 19.29) (see Fig. 5 A). These findings suggest that tACS may enhance episodic memory recall by modulating oscillatory activity linked to working memory and long-term retrieval.

### EEG results: theta power

Building on the observed group × time interaction in RT and accuracy, we analyzed qEEG data, focusing on theta power at F3 and F4—the sites of the anodal and cathodal electrodes—as well as at other frontal sites, F7 and F8, to help identify the primary frontal electrode associated with the stimulation-related effects. Theta power dynamics (mean absolute amplitude) were examined across WM-task processing events—encoding, active storage, and retrieval, separately—using the same modeling approach as for behavioral outcomes, incorporating group, time, and their interaction to explore patterns across the three time points.

The linear mixed-effects model was run with sham group at Time 1 as the reference condition of the dummy variable. Across all three task stages, the sham group showed no significant change at Time 2 but a robust increase in theta power at Time 3 (Encoding: *β* = 0.73, *p* <.001; Storage: *β* = 0.92, *p* <.001; Retrieval: *β* = 0.64, *p* <.001). No baseline differences were observed between active and sham groups at Time 1 in any stage.

The critical effects were found in the group × time interactions. At Time 2, the active group exhibited significantly lower theta power relative to sham across all stages (Encoding: *β* = − 0.96, *p* <.001; Storage: *β* = − 0.98, *p* <.001; Retrieval: *β* = − 0.89, *p* <.001). At Time 3, this relative reduction was even stronger, with marked decreases in the active group compared with sham (Encoding: *β* = − 1.31, *p* <.001; Storage: *β* = − 1.88, *p* <.001; Retrieval: *β* = − 1.43, *p* <.001).

**Fig. 4:**
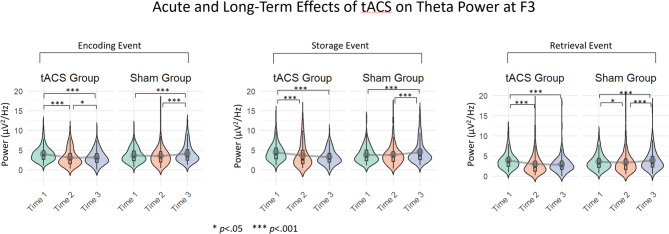
Theta power across three WM-task events (encoding, storage, and retrieval), grouped by group (tACS/Sham) and time points: baseline (T1 = Time 1), 5 days post-baseline (T2 = Time 2), 4 weeks post intervention (T3 = Time 3).

Pairwise comparisons within the active group with Bonferroni correction across time revealed a consistent decline in theta power across all task stages. During encoding, theta power was significantly reduced from Time 1 to Time 2 (β = 0.95, *p* <.001) and from Time 1 to Time 3 (β = 0.58, *p* <.001), with an additional decrease between Time 2 and Time 3 (β = − 0.37, *p* =.017). A similar pattern emerged during storage, where theta power dropped from Time 1 to both Time 2 (β = 0.88, *p* <.001) and Time 3 (β = 0.96, *p* <.001), while level remained stable between Time 2 and Time 3 (*p* = 1.00). Finally, during retrieval, theta power again declined from Time 1 to Time 2 (β = 1.01, *p* <.001) and from Time 1 to Time 3 (β = 0.79, *p* <.001), with no further reduction between Time 2 and Time 3 (*p* =.53) (Tables 4 and 5; Fig. 4).

At F4, F7, and F8, the trends in theta power did not align with the observed patterns in behavioral results. In the active group, theta power increased significantly from Time 1 to Time 2 across all phases, (*p* <.01; *p* <.01; *p* <.05) peaking mid-task, and then declined at Time 3 to a level close to baseline (all *p*s = 1.0). In contrast, in the sham group, theta power exhibited a consistent upward trend across all phases, steadily increasing relative to baseline, with a statistically significant rise from Time 1 to Time 3 (all *p*s < 0.001).

In summary, at F3, the active group exhibited a significant decline in theta power across all phases from Time 1 to Time 3, indicating a sustained left-prefrontal theta-power reduction relative to baseline. While a slight recovery or stabilization of mean theta power was observed in some phases between Time 2 and Time 3, it remained significantly below baseline levels, highlighting the enduring impact of the stimulation on neural oscillatory activity. In contrast, the sham group followed a non-linear trajectory, with a slight initial decrease followed by a pronounced recovery by the third time point, often exceeding baseline levels. This pattern may reflect natural compensatory processes or non-specific effects over time, highlighting the distinct differences in neural responses between the two groups.

**Table 4:** Estimated Means, standard Errors, and confidence intervals of theta power across three WM-task events (encoding, storage, and retrieval), grouped by group (tACS/Sham) and time points: baseline (Time 1), 5 days post-baseline (Time 2), and 4 weeks post intervention (Time 3).

Event	Group	Time	E.mean	SE	df	Lower.CL	Upper.CL
Encoding	Active	Time 1	3.95	0.351	31.4	3.24	4.67
		Time 2	3.00	0.35	31.1	2.29	3.71
		Time 3	3.37	0.349	30.9	2.66	4.08
	Sham	Time 1	3.72	0.35	31.3	3.01	4.44
		Time 2	3.42	0.349	30.8	2.71	4.14
		Time 3	4.48	0.35	31.3	3.77	5.2
Active storage	Active	Time 1	4.38	0.419	30.9	3.53	5.24
		Time 2	3.51	0.418	30.6	2.65	4.36
		Time 3	3.43	0.417	30.4	2.58	4.28
	Sham	Time 1	3.95	0.419	30.8	3.10	4.81
		Time 2	3.70	0.417	30.2	2.85	4.55
		Time 3	4.84	0.419	30.8	3.99	5.7
Retrieval	Active	Time 1	3.87	0.3	34.5	3.26	4.48
		Time 2	2.86	0.298	33.8	2.26	3.47
		Time 3	3.08	0.297	33.3	2.48	3.69
	Sham	Time 1	3.58	0.3	34.2	2.98	4.19
		Time 2	3.16	0.297	33.1	2.55	3.76
		Time 3	4.26	0.3	34.3	3.65	4.87

**Table 5:** Fixed effects for theta power at F3, with marginal R² (m. R²) and conditional R² (c. R²).

	Fixed effects	Estimate	SE	df	t value	Pr(>|t|)	m. *R*²	c. *R*²
	(Intercept)	3.638	0.3517	31.08	10.345	< 0.001 ***		
Encoding	Active	0.315	0.4957	30.69	0.636	0.530		
	Time2	0.009	0.1322	1961.0	0.068	0.946	0.397	0.39
	Time3	0.728	0.1357	1964.0	5.368	< 0.001 ***		
	Active X Time2	−0.961	0.1898	1962.0	−5.064	< 0.001 ***		
	Active X Time3	−1.311	0.191	1964.0	−6.866	< 0.001 ***		
Storage	(Intercept)	3.8711	0.4238	30.435	9.134	< 0.001 ***		
	Active	0.5128	0.5997	30.5043	0.855	0.399		
	Time2	0.104	0.1544	1973.4028	0.674	0.501	0.055	-
	Time3	0.9245	0.1584	1978.085	5.838	< 0.001 ***		
	Active X Time2	−0.9816	0.2217	1975.9168	−4.427	< 0.001 ***		
	Active X Time3	−1.8802	0.223	1977.792	−8.431	< 0.001 ***		
Retrieval	(Intercept)	3.5072	0.3016	33.8674	11.627	< 0.001 ***		
	Active	0.3648	0.4267	33.9163	0.855	0.399		
	Time2	−0.1128	0.159	1954.5565	−0.709	0.478	0.246	0.035
	Time3	0.6405	0.1631	1962.3894	3.928	< 0.001 ***		
	Active X Time2	−0.8948	0.228	1956.9416	−3.924	< 0.001 ***		
	Active X Time3	−1.4313	0.2293	1961.1557	−6.242	< 0.001 ***		

### Theta-WM relationships

To further investigate the associations between theta power and WM, we analyzed correlations between changes in theta power and task performance to determine whether these modulations are functionally linked to the observed improvements in WM RTs, WM accuracy, and explicit recall. Specifically, we examined three types of changes in theta power across the three time points of WM performance: from time 1 to time 2 (ΔTheta 2 − 1), from time 1 to time 3 (ΔTheta 3 − 1), and from time 2 to time 3 (ΔTheta 3 − 2) within the three task events: encoding, active storage, and retrieval. These Δs were analyzed in relation to corresponding changes in RT (ΔRT) and accuracy (ΔACC).

**Fig. 5:**
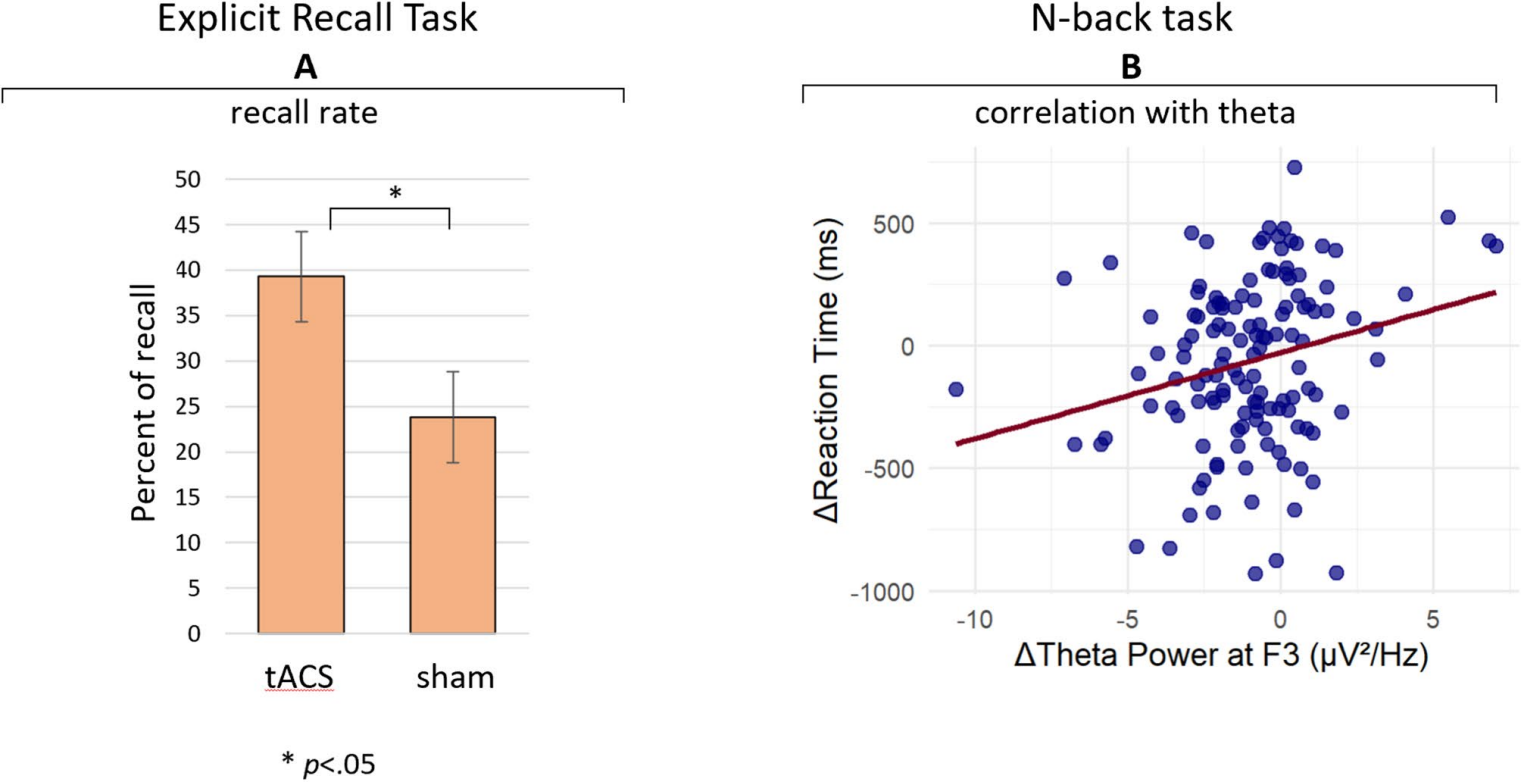
Panel **A**: Mean percentage of recall during encoding events (two-word displays within the WM task) of time 3 (4 weeks post stimulation) group. Panel **B**: The mean differences (Δs) in theta power at the F3 electrode correlate with WM reaction times (RTs) from Time 1 to Time 3 (Δ3 − 1) during retrieval events. Each dot represents a correct retrieval trial from an individual participant, taken from the long-format dataset in which each row corresponds to a single trial per participant. The correlation analysis included trials that were available across all three time points.

For the active group, the correlation analysis revealed associations between changes in theta power and RT in specific task phases. In both the encoding and active storage phases, a marginally significant positive correlation was observed between ΔRT 3 − 2​ and ΔTheta 3 − 2​ (*r* =.16, *p* =.05 for each phase). Importantly, in the retrieval phase, a significant correlation was found between ΔRT 3 − 1 and ΔTheta 3 − 1 (*r* =.24, *p* =.004) (see Fig. 5B). Additionally, a trend-level association was observed between ΔRT 3 − 2​ and ΔTheta 3 − 2​ (*r* =.15, *p* =.06) in retrieval, further suggesting that sustained theta changes could influence retrieval-related RT over time. However, for the changes from time 1 to time 2, no meaningful correlations were found, indicating that short-term changes in theta power immediately following stimulation were not directly linked to RT performance. In contrast, in the sham group no significant correlations were observed, further emphasizing the distinctiveness of neural-behavioral relationships in the active group. For accuracy, no significant correlations were observed between theta power deltas and the corresponding accuracy deltas.

For the explicit recall task, conducted exclusively at 4 weeks post intervention, correlations were analyzed between recall performance and theta power across the three task phases at this time point. In the active group, theta power demonstrated varying degrees of association with recall success. The strongest and most significant relationship was observed during the encoding phase (*r* = −.58, *p* =.02), where lower theta power at 4 weeks post intervention was linked to better recall. In the retrieval phase, a marginally significant negative correlation (*r* = −.49, *p* =.08) indicated that reduced theta power during retrieval may support successful memory retrieval. The active storage phase showed a weaker, non-significant trend (*r* = −.41, *p* =.15), suggesting a less pronounced role in delayed recall performance at this stage. No correlations were found in the sham group, suggesting that the observed relationships are specific to the active stimulation condition.

## Discussion

In support of our hypothesis, the current findings demonstrate that WM-related frontal theta activity can be significantly impacted by 6-Hz multi-session tACS targeting the left and right DLPFC, immediately after the intervention, and at four-weeks post tACS intervention. More so, long-term WM enhancement and higher episodic memory accuracy four weeks after intervention was observed only in the active tACS group. Long-term behavioral effects were accompanied by significant changes in WM-related frontal theta activity and in WM RT’s baseline to 4 weeks post tACS intervention. The acute and long-term effect on frontal theta activity under left prefrontal F3 electrode observed during encoding, active storage, and retrieval WM events supports the increased sensitivity of WM-related left prefrontal theta activity (mean theta power across 2000 ms windows) in identifying bifrontal theta-tACS effects on verbal WM brain-related activity. The discrete long-term effects on WM performance and theta activity are likely a reflection of long-term plasticity effects associated with fine-tuning functional frontal theta activity and episodic memory formation. More so, tACS improved WM retrieval at four weeks post intervention, indicating more efficient executive-attention DLPFC-network involvement, and efficient functional connectivity with medial-temporal lobe (MTL) regions, which are likely to improve communication with posterior parietal cortex (PPC) regions during WM retention periods^10,12,50–52^.

Importantly, our hypotheses regarding acute and long-term effects of multi-session bifrontal theta-tACS on verbal WM performance, explicit recall, and frontal theta oscillations in a double-blind randomized sham-controlled trial were partially supported. Mainly, the significant long-term improvement in WM RT’s and at four-weeks post intervention, and the observed reduction in left prefrontal theta power immediately and at four-weeks post tACS intervention, confirm the research predictions regarding the positive impact of theta-tACS on WM functioning, and particularly over a 4-week delay, following the intervention period. Furthermore, memory-circuitry models in animals and humans suggest that long-term plasticity effects may have been driven by prefrontal-MTL theta-synchrony synaptic-changes^39,53^, tentatively supported by explicit recall enhancement and left-prefrontal theta (lPFCt) power reduction across all WM events in the active tACS versus sham group and at four-weeks post tACS intervention. Importantly, the sham-tACS group showed the opposite effect after four weeks. Hence, their lPFC mean theta power significantly increased at four-weeks post-intervention. The excessive theta power after-effects found in the sham stimulation group at four-weeks post intervention could indicate increased but less efficient PFC recruitment during WM retrieval events^14^. Specifically, the reduction in WM RT’s at post four-weeks from baseline were related to reduced lPFCt power during WM retrieval events. In correspondence, excessive lPFCt power during WM encoding events was found to be related to reduced explicit recall accuracy at four-week post intervention. These consistent WM-related qEEG findings indicate a clear association between prefrontal theta power decrements and improved WM performance. This finding suggests that changes in prefrontal theta activity may be sensitive to early sustained changes in neural plasticity that result in long-term memory changes and support enhanced WM functioning, as observed following a four-week post-intervention period.

Frontal theta oscillations are crucial for decision-making, particularly in processing errors and modifying goal-directed WM responses via the anterior cingulate cortex^54,55^. Suppression of frontal theta power during incorrect WM responses predicts lower confidence and performance^54^. Frontal midline theta also plays a key role in maintaining temporal order information in WM^6^. In the current study, active tACS improved WM recognition both acutely and after a four-week delay. Participants in the active group also showed enhanced episodic memory consolidation, with explicit recall after-effects that indicate long-term plasticity supported by hippocampal-network long-term potentiation^56^. Interestingly, these behavioral benefits were accompanied by reduced theta power during WM encoding and retrieval in the active tACS group. Although many studies link increased frontal theta to better WM performance, evidence from Pahor & Jaušovec^24^ demonstrates that theta-tACS does not uniformly increase theta power; under some conditions, it may even reduce it. They reported that during sham sessions theta amplitude increased after stimulation, whereas during theta-tACS sessions theta amplitude decreased, and these effects varied by stimulation site and electrode location. This suggests that theta modulation is not straightforwardly additive, and reduced frontal theta after stimulation may reflect more efficient neural dynamics supporting WM maintenance and memory consolidation. Moreover, Aktürk et al.^17^ demonstrated that decreasing the individual theta frequency by 1 Hz via tACS enhanced visual memory capacity, supporting models of theta–gamma coupling in which slower theta cycles allow more gamma cycles to be nested, thereby expanding memory capacity. In line with this interpretation, we propose that the observed theta suppression in the active group reflects more efficient network processing, either by preventing maladaptive over-synchronization or by promoting reorganization of hippocampo–prefrontal coupling, thereby supporting stronger WM and LTM performance despite lower local theta power.

Regarding changes in WM functioning over time, active tACS has induced differential time-dependent changes in WM accuracy versus WM RTs. WM accuracy improved from baseline to four-week after intervention termination and was higher at 4 weeks after intervention than accuracy measured immediately after intervention. However, WM RTs were significantly reduced only from baseline to four-weeks post intervention. Regarding accuracy and RTs not affected by the intervention, WM accuracy and RTs were unchanged immediately after intervention versus baseline in both groups. The most robust effect on overall WM performance was observed at four-weeks post intervention. Therefore, significant long-term effects on accuracy and RT’s versus baseline imply that the current prefrontal tACS intervention may have modulated prefrontal theta-based neuroplasticity associated with WM performance following a four-week delay, which represents synchronicity changes in theta-related WM mechanisms with access to hippocampal encoding networks^53,56^. These long-term memory encoding networks are affected by disengagement of the default mode network (DMN) subnetwork involving the ACC and the medial prefrontal cortex (mPFC). In different WM task events (e.g., encoding events versus retrieval events), the dorsolateral prefrontal cortex (dLPFC) differentially active, which represents an executive attention network allowing access to target information towards immediate memory recognition. Increasing PFC connectivity with semantic cortical networks seems to increase theta–gamma cross frequency coupling to support multimodal-sensory-maintenance in WM, resulting in faster correct WM responses^6,12,14,39^. Together, these findings imply a close link between long-term hippocampal-PFC modulation of WM networks (possibly driven by CA1 cells in the hippocampus) and improved episodic memory recall following prefrontal-cortex theta-tACS intervention in healthy adult humans.

A number of research limitations that should be mentioned. One limitation of this study is the relatively small sample size per research group (*n* = 15), which restricts the generalizability of the findings to broader populations. The second limitation is that we tested episodic memory performance only 4 weeks post stimulation, restricting the conclusion regarding theta tACS effects on WM performance immediately following the five stimulation sessions. Additionally, it is difficult to determine the neural generators of theta power changes, thus, employing fMRI in future studies could reveal significant BOLD signal changes in specific brain regions (PFC and hippocampus), and changes in PFC connectivity, associated with tACS effects on WM functioning and explicit memory recall^12^.

Overall, the current findings demonstrated that bifrontal theta-tACS led to verbal WM performance enhancement and more accurate episodic memory retrieval at different post tACS time-points. The observed benefits of active tACS at PFC are likely due to theta-mediated synaptic plasticity changes within the prefrontal cortex-hippocampal networks^6^. To confirm long-term changes in synaptic function, further intracranial recordings at target prefrontal networks in animals and humans (epileptic syndrome patients) is needed. Magnetoeneceplagraphy (MEG) data obtained non-invasively for humans can be helpful to support these neural network activation changes as well associated with changes in neural plasticity.

In sum, these effects underscore the role of prefrontal theta oscillations in WM retrieval and point to possible contributions to episodic memory recall. These findings may pave the way for developing focal tACS techniques to enhance cognitive function in healthy individuals, and in clinical populations suffering from WM impairments and PFC dysfunction.

## Data Availability

The datasets used and/or analysed during the current study available from the corresponding author on reasonable request.
